# Trend-TDT – a transmission/disequilibrium based association test on functional mini/microsatellites

**DOI:** 10.1186/1471-2156-8-75

**Published:** 2007-11-01

**Authors:** Bing-Jian Feng, David E Goldgar, Marilys Corbex

**Affiliations:** 1 Department of Dermatology, University of Utah School of Medicine, Salt Lake City, USA; 2 Eastern Mediterranean Regional Office, World Health Organization, Cairo, Egypt

## Abstract

**Background:**

Minisatellites and microsatellites are associated with human disease, not only as markers of risk but also involved directly in disease pathogenesis. They may play significant roles in replication, repair and mutation of DNA, regulation of gene transcription and protein structure alteration. Phenotypes can thus be affected by mini/microsatellites in a manner proportional to the length of the allele. Here we propose a new method to assess the linear trend toward transmission of shorter or longer alleles from heterozygote parents to affected child.

**Results:**

This test (trend-TDT) performs better than other TDT (Transmission/Disequilibrium Test) type tests, such as TDT_max _and TDT_L/S_, under most marker-disease association models.

**Conclusion:**

The trend-TDT test is a more powerful association test when there is a biological basis to suspect a relationship between allele length and disease risk.

## Background

Variable number tandem repeats (VNTR's) are repetitive DNA sequences widely dispersed in the human genome. They are highly unstable and thus display a remarkable degree of polymorphism. They vary in length from a few to several thousand nucleotides and vary in complexity from simple di-, tri- and tetra-nucleotide repeats (microsatellites) to more complex repetitive elements (minisatellites). VNTR's, mainly microsatellites, have assumed an increasingly important role as markers in the genome and are intensively exploited for gene mapping. But VNTR's could be associated with human disease, not only as markers but also directly involved in disease pathogenesis; indeed, several functions have been suggested for micro- and mini-satellite DNA sequences.

If located within a coding sequence, VNTR's may alter protein structure. For example, expansions of tri-nucleotide microsatellites are responsible for genetic diseases such as X-linked spinal and bulbar muscular atrophy, Huntington disease, type 1 spinocerebellar ataxia, dentatorubral-pallidoluysian atrophy, and Machado-Joseph disease. These diseases are caused by expansion of CAG triplets within protein-coding regions [1].

VNTR's may also regulate gene transcription. Numerous *in vitro *studies have shown that gene transcription may be increased or decreased proportionally to the number of repeated sequences (i.e. length of alleles) as illustrated in Table 1 (for detailed review, see Kashi et al. [2]). Direct effect of transcriptional modulation on risk of disease has been observed. As an example, the minisatellite *ILPR *(Insulin-Linked Polymorphic Region, (ACAGGGGTGTGGGG)_n_) located 5' of the Insulin gene is implicated in Insulin-Dependent Diabetes Mellitus [3]. To date, many transcriptional factors have been identified and their binding with minisatellite repeated sequences have been demonstrated. There is increasing evidence that some gene-disease associations are due to functional micro/minisatellites, with the magnitude of susceptibility being related to allele length [4-6].

**Table 1:** Micro/minisatellites that regulate gene transcription.

Genes regulated	VNTR localization	Repeat unit	Length of alleles	Transcription regulation ^a^	Interacting factor ^b^
**Microsatellites**					
EGF receptor	intron 1	(CA)_n_	14–21	down	
metalloproteinase 9	promoter	(CA)_n_	14–23	down	
Pax-6	promoter -1 kb	(AC)_m _(AG)_n_	24–36	up	
HLA-DRB	intron 2	(GT)_m _(GA)_n_	(15–22)(4–15)	down	CTCF (ZNF)
NOS2A	promoter -2.5 kb	(CCTTT)_n_	8–18	up	
COL1A2	promoter, intron 1	(CA)_n_(CG)_n_(CA)_n_	(14–21)(6–7)(8)	up	
prolactin	promoter	(TG)_m _(CA)_n_	(8–15)(4–13)	down	
phospholipase A2	promoter -595 bp	(CA)_n_	48	down	
heme oxygenase	promoter -240 bp	(GT)_n_	16–38	up	
CD30 gene	promoter -400 bp	(CCAT)_n_	2–12	repression	
**Minisatellites**					
HRAS	3':1 kb after polyA	28 bp repeat	30–84	up	NF-kappa B
Insulin	promoter -596 bp	14 bp repeat	40–157	up	Pur-1
ABO gene	promoter -3.6 kb	43 bp repeat	4–6	up	CBF/NF-Y

^a ^The direction of transcription regulation is according to elongation of alleles;

^b ^If identified, transcription factors that interact with the micro/minisatellites are mentioned.

The Transmission/Disequilibrium Test (TDT) is a popular method to assess the involvement of a candidate gene or a genome region in the genetic component of a disease, using cases and their parents. The TDT, as originally developed [7], tested the association between a bi-allelic marker and a disease. Many authors have proposed an extension of the TDT to multi-allelic markers, by testing each allele separately [8,9]., by testing symmetry of the transmitted/non-transmitted table [10,11], by testing marginal homogeneity [12,13], or by conditional logistic regression [14,15]. However, all these extensions considered implicitly the multi-allelic marker as a polymorphism without function, that is, the risk of disease was not treated as being correlated with allele repeat length. While this is true for most situations, there are some situations where the multi-allelic marker under study may have a functional effect on the studied disease, and thus this correlation may be present. This may introduce new information that can be taken into account in the test. From a statistical point of view, increased allele length could be understood as an increased dose of exposure to a risk factor. In contrast to case-control association studies where one can use the classical trend-chi-square (the Cochran-Armitage trend test) to test this hypothesis, available extensions of the TDT to multi-allelic markers do not test such a "dose effect" in family-based association studies. However, case-control studies can be subject to bias produced by hidden population stratification. Therefore, a new statistical method that can test the correlation of allele length with disease susceptibility, and is not sensitive to population stratification is needed. In this paper, we describe a newly developed method to meet this requirement.

## Methods

### Algorithm

Consider a multi-allelic marker with *k *alleles, which are assumed to be coded as integers proportional to their length. The trend-TDT statistic is based on the length of alleles transmitted from heterozygous parents to their affected children. Let's denote, for each heterozygous parent *i*, *t*_*i *_the length of the transmitted allele, *u*_*i *_the length of the untransmitted allele, and *x*_*i *_the difference between the length of transmitted and untransmitted alleles (*x*_*i *_= *t*_*i*_*-u*_*i*_). For family *f*, let *n*_*f *_be the number of calculated *x*_*i *_within the family, and define *d*_*f *_as

df=∑xinf
 MathType@MTEF@5@5@+=feaafiart1ev1aaatCvAUfKttLearuWrP9MDH5MBPbIqV92AaeXatLxBI9gBaebbnrfifHhDYfgasaacPC6xNi=xI8qiVKYPFjYdHaVhbbf9v8qqaqFr0xc9vqFj0dXdbba91qpepeI8k8fiI+fsY=rqGqVepae9pg0db9vqaiVgFr0xfr=xfr=xc9adbaqaaeGacaGaaiaabeqaaeqabiWaaaGcbaGaemizaq2aaSbaaSqaaiabdAgaMbqabaGccqGH9aqpjuaGdaWcaaqaamaaqaeabaGaemiEaG3aaSbaaeaacqWGPbqAaeqaaaqabeqacqGHris5aaqaamaakaaabaGaemOBa42aaSbaaeaacqWGMbGzaeqaaaqabaaaaaaa@387D@

Under the situation that neither the micro/minisatellite is the cause of the disease, nor is it in linkage disequilibrium with any disease causing genes, then the mean of *d*_*f *_should be zero, and its variance is

V(df)=V(∑xinf)=V(∑xi)nf=nfV(x)nf=V(x)
 MathType@MTEF@5@5@+=feaafiart1ev1aaatCvAUfKttLearuWrP9MDH5MBPbIqV92AaeXatLxBI9gBaebbnrfifHhDYfgasaacPC6xNi=xI8qiVKYPFjYdHaVhbbf9v8qqaqFr0xc9vqFj0dXdbba91qpepeI8k8fiI+fsY=rqGqVepae9pg0db9vqaiVgFr0xfr=xfr=xc9adbaqaaeGacaGaaiaabeqaaeqabiWaaaGcbaGaemOvayLaeiikaGIaemizaq2aaSbaaSqaaiabdAgaMbqabaGccqGGPaqkcqGH9aqpcqWGwbGvcqGGOaakjuaGdaWcaaqaamaaqaeabaGaemiEaG3aaSbaaeaacqWGPbqAaeqaaaqabeqacqGHris5aaqaamaakaaabaGaemOBa42aaSbaaeaacqWGMbGzaeqaaaqabaaaaOGaeiykaKIaeyypa0tcfa4aaSaaaeaacqWGwbGvcqGGOaakdaaeabqaaiabdIha4naaBaaabaGaemyAaKgabeaaaeqabeGaeyyeIuoacqGGPaqkaeaacqWGUbGBdaWgaaqaaiabdAgaMbqabaaaaOGaeyypa0tcfa4aaSaaaeaacqWGUbGBdaWgaaqaaiabdAgaMbqabaGaemOvayLaeiikaGIaemiEaGNaeiykaKcabaGaemOBa42aaSbaaeaacqWGMbGzaeqaaaaakiabg2da9iabdAfawjabcIcaOiabdIha4jabcMcaPaaa@5BE0@

Note that this *d*_*f *_is actually the mean of *x*_*i *_weighted by square root of *n*_*f*_, so that the variance of *d*_*f *_is equal between families. Hence the test statistic

T=mean(df)S/N
 MathType@MTEF@5@5@+=feaafiart1ev1aaatCvAUfKttLearuWrP9MDH5MBPbIqV92AaeXatLxBI9gBaebbnrfifHhDYfgasaacPC6xNi=xI8qiVKYPFjYdHaVhbbf9v8qqaqFr0xc9vqFj0dXdbba91qpepeI8k8fiI+fsY=rqGqVepae9pg0db9vqaiVgFr0xfr=xfr=xc9adbaqaaeGacaGaaiaabeqaaeqabiWaaaGcbaGaemivaqLaeyypa0tcfa4aaSaaaeaacqWGTbqBcqWGLbqzcqWGHbqycqWGUbGBcqGGOaakcqWGKbazdaWgaaqaaiabdAgaMbqabaGaeiykaKcabaGaem4uamLaei4la8YaaOaaaeaacqWGobGtaeqaaaaaaaa@3C1F@

asymptotically follows the Student's t distribution with *N-1 *degrees of freedom. Here *S *is the estimated standard deviation of the *d*_*f*_, and *N *is the number of informative families. In case there is a trend toward transmission of shorter alleles, the *mean(d*_*f*_*) *will be less than 0, and vice versa. If biological clues indicate that preferential transmission of shorter alleles (or longer alleles) should be observed, the test is one-tailed t test (H_1_:*T *< 0 or H_1_:*T *> 0); otherwise the test is two-tailed (H_1_: *T *≠ 0).

The missing genotype problem is treated according to Curtis [16]. In case both parents are missing, or, one parent is missing and the affected child has the same heterozygote genotype as the other parent, these families are considered uninformative and are discarded in the analysis. When only one parent is missing but the affected child is homozygote, inclusion of such triads will lead to bias, therefore they are also discarded [16]. In other situations, transmission status of either allele can be inferred, and they are used in the analysis.

### Comparison with other methods

Two other methods that can be used in testing association between disease and functional micro/minisatellites are TDT_max _and TDT_L/S_. TDT_max _stems from the classical bi-allelic TDT. The statistics corresponding to TDT_max _is the maximum chi-square value obtained over all alleles:

TDTmax⁡=max⁡i(ni•−n•i)2(ni•+n•i)(i=1...k)
 MathType@MTEF@5@5@+=feaafiart1ev1aaatCvAUfKttLearuWrP9MDH5MBPbIqV92AaeXatLxBI9gBaebbnrfifHhDYfgasaacPC6xNi=xI8qiVKYPFjYdHaVhbbf9v8qqaqFr0xc9vqFj0dXdbba91qpepeI8k8fiI+fsY=rqGqVepae9pg0db9vqaiVgFr0xfr=xfr=xc9adbaqaaeGacaGaaiaabeqaaeqabiWaaaGcbaqbaeqabeGaaaqaaiabdsfaujabdseaejabdsfaunaaBaaaleaacyGGTbqBcqGGHbqycqGG4baEaeqaaOGaeyypa0ZaaCbeaeaacyGGTbqBcqGGHbqycqGG4baEaSqaaiabdMgaPbqabaqcfa4aaSaaaeaacqGGOaakcqWGUbGBdaWgaaqaaiabdMgaPjabgkci3cqabaGaeyOeI0IaemOBa42aaSbaaeaacqGHIaYTcqWGPbqAaeqaaiabcMcaPmaaCaaabeqaaiabikdaYaaaaeaacqGGOaakcqWGUbGBdaWgaaqaaiabdMgaPjabgkci3cqabaGaey4kaSIaemOBa42aaSbaaeaacqGHIaYTcqWGPbqAaeqaaiabcMcaPaaaaOqaaiabcIcaOiabdMgaPjabg2da9iabigdaXiabc6caUiabc6caUiabc6caUiabdUgaRjabcMcaPaaaaaa@5C52@

Here *n*_*i*• _denote the number of heterozygote parents who transmit an allele *i*, and *n• *_*i *_denote the number of heterozygote parents who has an allele *i *but do not transmit it. Individual TDT is calculated for all alleles, and the maximal value is taken as the TDT_max_. Although the individual TDT test follows Chi-square distribution with 1 degree of freedom, the TDT_max _does not. Clearly, this method will not have appropriate type I error due to the selection of the highest Chi-square value. Several methods have been proposed to address the multiple testing problem in TDT_max_, including empirical p value simulation [9] and modified Bonferroni correction [8]. Since the former method requires enormous number of repetitions to accurately obtain a low p value, in this study, Bonferroni corrected TDT_max _is used and evaluated.

TDT_L/S _corresponds to the classical bi-allelic TDT computed on collapsed long alleles vs. collapsed short alleles. In this case, the traditional TDT statistics can be used:

TDTL/S=(b−c)2b+c
 MathType@MTEF@5@5@+=feaafiart1ev1aaatCvAUfKttLearuWrP9MDH5MBPbIqV92AaeXatLxBI9gBaebbnrfifHhDYfgasaacPC6xNi=xI8qiVKYPFjYdHaVhbbf9v8qqaqFr0xc9vqFj0dXdbba91qpepeI8k8fiI+fsY=rqGqVepae9pg0db9vqaiVgFr0xfr=xfr=xc9adbaqaaeGacaGaaiaabeqaaeqabiWaaaGcbaGaemivaqLaemiraqKaemivaq1aaSbaaSqaaiabdYeamjabc+caViabdofatbqabaGccqGH9aqpjuaGdaWcaaqaaiabcIcaOiabdkgaIjabgkHiTiabdogaJjabcMcaPmaaCaaabeqaaiabikdaYaaaaeaacqWGIbGycqGHRaWkcqWGJbWyaaaaaa@3E71@

where *b *is the number of parents that transmit the long allele but not the short one, and *c *is the number of parents that transmit the short allele but not the long one. It should be noted that some of the heterozygote parents are not counted in the computation if both of their alleles belong to the long allele pool or short allele pool. The specific problem of this approach is the choice of the threshold between "long" and "short" alleles; here we choose the first allele (from shortest to longest) whose cumulative allele frequency is greater than 0.5, so that roughly half of the alleles are long alleles and another half the short ones. We note however that in some cases there be relevant biological data which might suggest a more appropriate threshold.

The cut-of thresholds to reject H_0 _hypothesis used in these two methods are the same as trend-TDT.

### Type I error computations

In order to assess and compare the type I error rates of each of the three tests, we simulated 200 trios (case and both parents) with disease-unrelated microsatellite genotypes. The total number of alleles of this marker is set to 10, with equal allele frequencies. Simulations are performed 1,000,000 times. The proportion of times that calculated p-value is equal to or less than an expected value is plotted against this expected value, in minus logarithm scale. For a correct test statistic, this curve should be exactly the line "y = x". For a test with higher type I error rate, the curve will be bellow the line "y = x", and for a conservative test, the curve lies above.

### Modeling genotyping errors

The most common genotyping errors in microsatellites were simulated to evaluate their effects on type I error rate of the trend-TDT test. These errors include confusing homozygote and adjacent-allele-heterozygote genotypes in allele banding pattern scoring [17], false homozygotes due to the preferential amplification of shorter alleles over longer alleles (short allele dominance), false homozygotes due to priming site mutations (null allele), offspring gaining one more repeat unit in one of the alleles (microsatellite mutation), and randomly mis-scoring an allele as its adjacent allele due to binning error. In simulation, each of these genotyping error rates was moderately higher than what is usually discovered in real data [18]. The microsatellite was simulated with 10 equally distributed alleles, without association with disease. Type I error rates were then calculated as the proportion of times trend-TDT yielding significant results (p ≤ 0.05) from 1,000,000 simulations on 200 trios.

### Power computations

Power can be estimated by generating samples with a determined pattern of marker-disease association, and by calculating the proportion of these simulations that the null hypothesis is correctly rejected. Here in this paper, we assume a significance level of 0.001. Following this design, we evaluate the power of the trend-TDT and compare it with the power of two other TDT tests: TDT_max _and TDT_L/S_.

The powers of the three tests were evaluated under different patterns of marker-disease association, parameterized in terms of relative-risk, and under different kinds of multi-allelic markers in terms of the number of alleles and allele frequencies. The different models are presented in Table 2. In these models, the maximum relative risk for any single allele size is always equal to 3, and the prevalence of the disease is fixed at 10%. Calculation of genotype-wide penetrance is based on multiplicative model. All estimates of power were based on 10,000 generated tests on 200 trios, unless otherwise specified.

**Table 2:** Alleles frequencies and allelic relative risks in power simulation.

Designation	Allele length						Notes
		
	1	2	3	4	5	6	
**Allele Frequencies**							
F6.eq	1/6	1/6	1/6	1/6	1/6	1/6	Equal allele frequencies.
F6.rd	.15	.20	.10	.40	.10	.05	Randomized allele frequencies.
F6.bi	.10	.10	.30	.30	.10	.10	There exist two major alleles.
**Relative Risk**							
RR(lin)	1	1.4	1.8	2.2	2.6	3	RRs increase linearly along with allele length.
RR(thr4) ^§^	1	1	1	3	3	3	RRs increase above a threshold of allele length.

^§ ^the number in the bracket denotes the first allele with higher risk.

### Modeling non-functional markers

Situations when VNTR markers are associated with a disease, without linear correlation between allele length and disease risk, are also modeled. In this model, the VNTR marker has 10 alleles, with allele frequencies equally distributed. Relative risks are assigned proportional to allele length, then before each repeat of the simulation, this relative risk vector is permuted. Empirical power is calculated to compare the performance of the statistics before and after permutation, based on 10,000 repeats of simulations on 200 trios.

A computer program for the trend-TDT, TDT_max_, and TDT_L/S _test is written and can be downloaded [19].

## Results

### Type I error

**Figure 1:**
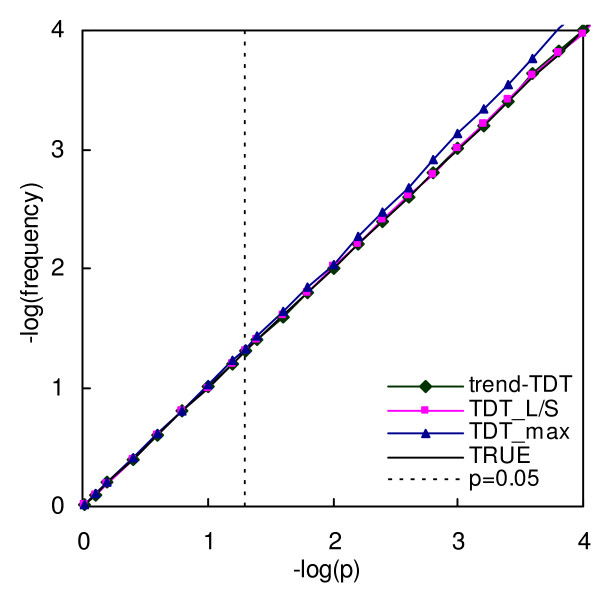
**Type I error rates for trend-TDT, TDT_max _and TDT_L/S_**. X axes is the expected p value in minus logarithm scale, Y axes is the observed frequencies that the calculated p value is equal to or less than the expected p value, in minus logarithm scale. The line "TRUE" is the expected curve for a correct test, which should be exactly the line "y = x".

As shown in Figure 1, the curve for both trend-TDT and TDT_L/S _are very close to the diagonal line, showing correct type I error rates in simulation. After Bonferroni correction, the type I error rate of TDT_max _is nearly correct, although it is still a little conservative. As shown in Table 3, genotyping errors lead to slightly inflated type I error rates for trend-TDT.

**Table 3:** Simulated genotyping errors and resultant type I error rates.

Error Models ^§^	Mistypes in total genotypes (%)	Misinheritance in mistyped trios (%)	Type I Err. (p ≤ 0.05) Rate (95% C.I.)
A	0.50	27	.0504 (.0499–.0508)
B	0.90	59	.0502 (.0498–.0506)
C	2.70	59	.0502 (.0497–.0506)
D	0.90	59	.0500 (.0496–.0505)
E	0.15	41	.0499 (.0495–.0503)
F	0.17	88	.0502 (.0498–.0507)
ALL	5.23	57	.0506 (**.0502–.0511**)

^§ ^**A**: mis-scoring genotype (x,x) as (x,x+2); **B**: mis-scoring genotype (x,x+2) as (x,x); **C**: short allele dominance; **D**: binning error; **E**: priming site mutation (Null allele); **F**: microsatellite mutation; **ALL**: all of above.

### Power

**Figure 2:**
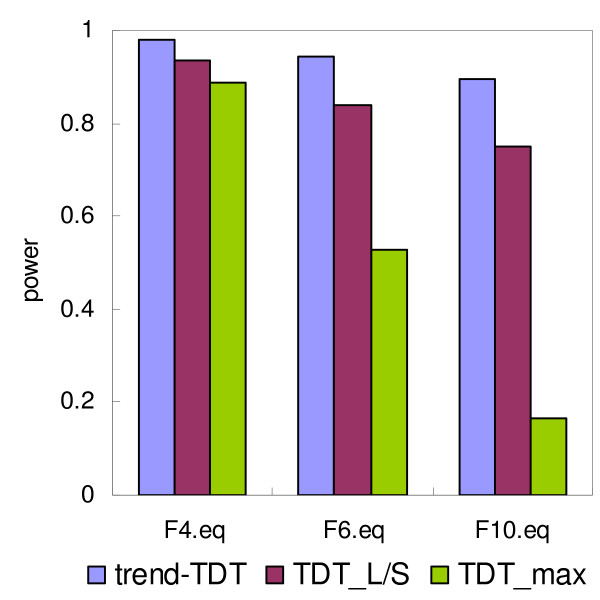
**Power of the TDT tests under different number of alleles**. Disease risk linearly increases along with the allele length. All allele frequencies are set to equal. Number of alleles are 4, 6 and 10, respectively.

**Figure 3:**
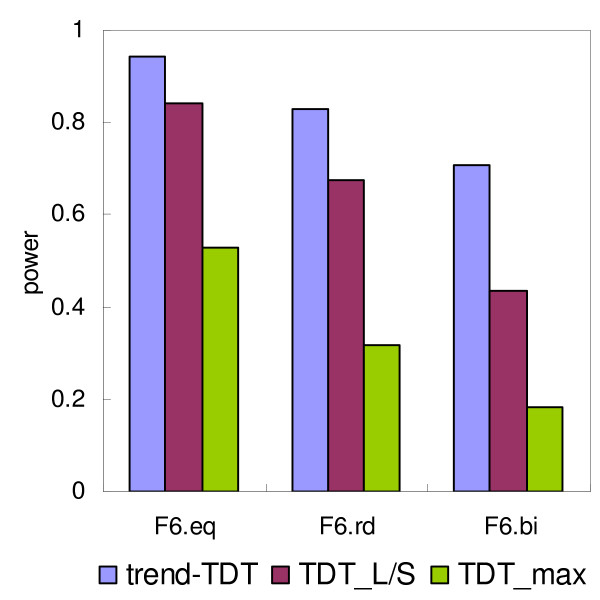
**Power of the TDT tests under different sets of allele frequencies**. Disease risk linearly increases along with the allele length, i.e. RR(lin) in Table 2. Number of alleles is set to 6. The allele frequencies are equal (F6.eq), random (F6.rd), or uneven, where two major alleles exist (F6.bi).

**Figure 4:**
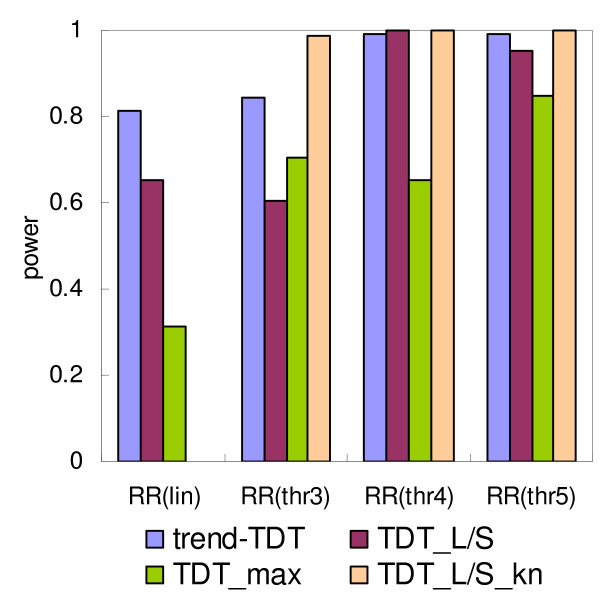
**Power of the TDT tests under different marker-disease association models**. RR(lin) designates the linear model, and RR(thr#) the threshold model, where # denotes the first allele with higher risk (Table 2). Number of alleles is set to 6, with equal allele frequencies, i.e. F6.eq in Table 2. "TDT_L/S" is the TDT_L/S _method using medium allele length as threshold, "TDT_L/S_kn" is the TDT_L/S _method when the threshold is known and is used in the test. Power is calculated from 10,000 simulations on 150 trios, using significant criteria 0.001.

The power of the three tests, trend-TDT, TDT_L/S _and TDT_max _on simulated trios are plotted in Figures 2, 3, 4. Figure 2 presents the power of the tests under different VNTR/STR models, which vary in terms of the number of alleles at the VNTR (4, 6 or 10 alleles with equal allele frequencies). In each of these models, the relative risk associated with each allele increases linearly with the length of the allele. The trend-TDT is clearly the most powerful test in all situations. An increase in the number of alleles resulted in decreased power for all tests; however, the trend-TDT was the least sensitive to this effect. Figure 3 presents the behavior of the tests under different sets of allele frequencies, assuming a linear relative risk model of the simulated functional VNTR. It can be seen from the figure that the power is higher when the allele frequencies are equally distributed, and is lower when some major alleles exist. This is probably related to the fact that overall heterozygosity (and thus informativeness of the sample) is maximized with equal allele frequencies. Nevertheless, the simulations indicate that the trend-TDT is the least sensitive to the distribution of allele frequencies and is the most powerful for association detection among the three methods.

The behavior of the tests under different marker-disease association models is presented in Figure 4. These models are defined so that relative risks increased linearly ("RR(lin)") or uniformly above a threshold ("RR(thr3)", "RR(thr4)", "RR(thr5)"), according to the increase in VNTR length. The assumed marker is a microsatellite with six equally frequent alleles. In the threshold models, the thresholds for higher relative risk are set to allele 3 ("RR(thr3)"), allele 4 ("RR(thr4)"), or allele 5 ("RR(thr5)"). As shown in Figure 4, the trend-TDT is the most powerful method under the linear model, while under threshold models, the relative performance depends on where the threshold is. When the threshold is close to the shortest or longest allele, the trend-TDT performed much better than TDT_L/S_. When the threshold is exactly in the middle, which is most favorable to TDT_L/S_, the TDT_L/S _is better. However, in this case both the trend-TDT and TDT_L/S _have high power and the difference is very small (Figure 4). If the threshold can be inferred by biologic knowledge of the gene under study, then using the known threshold will lead to much higher power in TDT_L/S _than the trend-TDT (Figure 4). Under most circumstances, TDT_max _performed the worst among the tested methods (Figure 2, 3, 4), with the only exception that in the RR(thr3) model in Figure 4, TDT_max _is better than TDT_L/S_.

**Figure 5:**
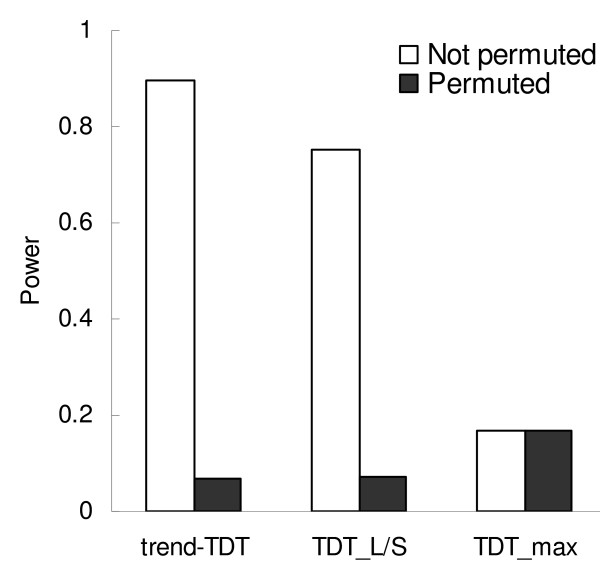
**Power of the TDT tests before and after permuting relative risk vector**. The disease model is linear relative risk of VNTR with 10 alleles.

When markers are associated with the studied trait, but without a specific trend, the power of TDT_max _remains unchanged, while the power of both the trend-TDT and TDT_L/S _decrease markedly (Figure 5). Notably, the trend-TDT and TDT_L/S _still have some power for association detection. In-depth study of each replicate of the simulation found that the power depends on the trend of the increase/decrease of the relative risk vector: in the most extreme cases where the trend is almost zero, the power of these two tests are equal to type I error rates; however, because in most cases, the trend is not zero, the power of trend-TDT and TDT_L/S _remain above the type I error level.

## Discussion

### Performance of the tests

As expected, when the relative risks increase proportionally with allele length, the trend-TDT is always more powerful than the other tests, irrespective of the number of alleles or their frequencies. When the RRs increase according to a threshold model, the performances of TDT_L/S _and trend-TDT depend on the threshold. TDT_L/S _is more sensitive to the threshold and less powerful when the threshold is close to the longest or shortest allele. When the threshold is close to medium allele length, TDT_L/S _performs slightly better than the trend-TDT, but both are quite powerful in this situation. The TDT_max _performs the worst in most situations studied here. This may be because both trend-TDT and TDT_L/S _use the information on the correlation between allele length and disease risk that is present in the generated disease model.

### Choice of the tests

Based on these results, we do not recommend the TDT_max _for any situation when there could be a relationship between allele length and disease risk. Whether to use trend-TDT or TDT_L/S _depends on prior knowledge of the functional relationship between allele length and gene function. When the threshold model is biologically true, and this threshold can be inferred by biologic knowledge of the gene under study, then TDT_L/S _is a better choice. Under all other situations, trend-TDT is recommended. When the threshold model is true but it is not clear where the threshold is, trend-TDT should be used, since by using TDT_L/S_, one either has a multiple testing problem by trying different thresholds, or alternatively has less power for the test by using the median allele length only, which could be wrong biologically. Even when the true threshold is close to the median allele length, the difference between trend-TDT and TDT_L/S _is so small that it could be ignored. In other situations when a VNTR is associated with a disease without trend, trend-TDT and TDT_L/S _are not as powerful, therefore other TDT methods should be used.

Another potential transmission/disequilibrium based test that could take into account the phenotypic response trend toward longer or shorter alleles is conditional logistic regression [20,21], using a continuous variable for the allele length rather than a categorical one. Preliminary simulations indicate that this test is not as powerful as the trend-TDT test (data not shown); nevertheless, conditional logistic regression could be more beneficial, since it can incorporate various genetic risk models, include other genetic or environmental risk factors, and provide estimates of the risk of the disease conferred by the functional micro/minisatellite. Therefore, both methods might be used depending on the particular study circumstances.

### Impact of genotyping errors

Given that genotyping errors may lead to increased type I error rates of TDT tests, several modified TDT statistics were proposed for analysis of single nucleotide polymorphisms [22-26], since it is much easier to model genotyping errors in bi-allelic markers than in multi-allelic markers. It was expected that genotyping errors would also increase the type I error rate of the trend-TDT test. However, simulation has shown that, with reasonable typing error frequencies, the type I error rates were inflated only slightly. The reason might be that genotyping errors in multi-allelic markers can be efficiently detected by Mendelian-inheritance analysis when parental data are available [27]. It should be noted that the extent of type I error is a function of the typing error frequencies, the number of alleles, the allele frequencies, and sample size [23,28]. Thus, if genotyping errors are observed in a subset of a larger sample of pedigrees (e.g., over 500 affected offspring), statistical methods to address genotyping errors in TDT analysis should be considered to confirm that significant results are not false positives due to undetected genotyping errors. To further eliminate genotyping errors in real data analysis, it is recommended that siblings of the patients are genotyped and/or closely adjacent markers are genotyped, so that more typing errors can be detected as either Mendelian inconsistencies in the former or haplotype double crossovers in the latter.

## Conclusion

In summary, we have developed a new statistical test, the trend-TDT test, appropriate for those situations when a) parental data are available; and b) there are multiple alleles at the marker locus hypothesized to be associated with the disease of interest; and, most importantly, c) there is a biological basis to suspect a relationship between allele length and disease risk.

## Authors' contributions

BJF carried out the programming, testing and simulation of the methods, and drafted the manuscript. DEG contributed to the design of the study and critical review of the manuscript, MC conceived the study, participated in its design and helped to draft the manuscript. All authors read and approved the final manuscript.
